# Introduction to the Special Issue: Advances in island plant biology since Sherwin Carlquist's *Island Biology*

**DOI:** 10.1093/aobpla/plv148

**Published:** 2015-12-31

**Authors:** Anna Traveset, José María Fernández-Palacios, Christoph Kueffer, Peter J. Bellingham, Clifford Morden, Donald R. Drake

**Affiliations:** 1 Mediterranean Institute for Advanced Studies (CSIC-UIB), C/Miquel Marqués 21, 07190 Esporles, Mallorca, Balearic Islands, Spain; 2 Island Ecology and Biogeography Research Group, Instituto Universitario de Enfermedades Tropicales y Salud Pública de Canarias, Universidad de La Laguna, La Laguna 38206, Tenerife, Spain; 3 Institute of Integrative Biology, ETH Zurich, Universitätstrasse 16, CH-8092 Zurich, Switzerland; 4 Landcare Research, PO Box 69040, Lincoln 7640, New Zealand; 5 Department of Botany, University of Hawai‘i at Mānoa, 3190 Maile Way, Honolulu, HI 96822, USA

**Keywords:** Biogeography, island ecology and conservation, oceanic islands, palaeoecology, phylogeography

## Abstract

Sherwin Carlquist's seminal publications—in particular his classic *Island Biology*, published in 1974—formulated hypotheses specific to island biology that remain valuable today. This special issue brings together some of the most interesting contributions presented at the First Island Biology Symposium hosted in Honolulu on 7–11 July 2014. We compiled a total of 18 contributions that present data from multiple archipelagos across the world and from different disciplines within the plant sciences. In this introductory paper, we first provide a short overview of Carlquist's life and work and then summarize the main findings of the collated papers. A first group of papers deals with issues to which Carlquist notably contributed: long-distance dispersal, adaptive radiation and plant reproductive biology. The findings of such studies demonstrate the extent to which the field has advanced thanks to (i) the increasing availability and richness of island data, covering many taxonomic groups and islands; (ii) new information from the geosciences, phylogenetics and palaeoecology, which allows us a more realistic understanding of the geological and biological development of islands and their biotas; and (iii) the new theoretical and methodological advances that allow us to assess patterns of abundance, diversity and distribution of island biota over large spatial scales. Most other papers in the issue cover a range of topics related to plant conservation on islands, such as causes and consequences of mutualistic disruptions (due to pollinator or disperser losses, introduction of alien predators, etc.). Island biologists are increasingly considering reintroducing ecologically important species to suitable habitats within their historic range and to neighbouring islands with depauperate communities of vertebrate seed dispersers, and an instructive example is given here. Finally, contributions on ecological networks demonstrate the usefulness of this methodological tool to advancing conservation management and better predicting the consequences of disturbances on species and interactions in the fragile insular ecosystems.

## Abstract (precis)

This is the introductory paper of the special issue emerging from the First Island Biology Symposium hosted in Honolulu on July 2014. The 18 papers compiled present data from multiple archipelagos across the world and from different disciplines within plant sciences, showing us how much island biology has advanced since Carlquist's inspiring contribution *Island Biology**.*** Such advance has been possible mostly thanks to the increasing availability and richness of island data, new information from the geosciences, phylogenetics, and palaeoecology, and new methodological advances in analyzing data at large spatial scales. A group of papers deal with issues to which Carlquist contributed most: long-distance dispersal, adaptive radiation, and plant reproductive biology, whereas others cover a range of topics related to plant conservation on islands.

## Introduction

In July 2014, the University of Hawai‘i hosted the First Island Biology Symposium, where nearly 450 island biologists from all over the world and from very different island biology subdisciplines (Island Biogeography, Phylogeography, Ecology, Palaeoecology, Conservation Biology, etc.) joined together for the first time (Kueffer *et al*. 2014). The aim of the event was to launch a regular series of International Island Biology conferences, bringing together researchers who, by the very nature of the islands they study, face geographical barriers to communication and collaboration. This event was broadly recognized as a big success and the Second Symposium, following the agreed policy of rotating the ocean of the archipelago hosting the next event, will be held in Angra do Heroismo, Azores, in summer 2016 (http://www.islandbiology2016.uac.pt/).

The year of the first Island Biology conference coincided with the 40th anniversary of the publication of an outstanding book that inspired a generation of island biologists: Sherwin Carlquist's *Island Biology*, published in 1974 by Columbia University Press. Unfortunately, unforeseen circumstances at the last minute prevented Sherwin Carlquist from attending the conference and presenting a keynote address.

This special issue brings together some of the most interesting botanical contributions presented at the Symposium. The 18 contributions that we compiled deal with very different themes and come from very different archipelagos across the world and disciplines within plant science. Indeed, the broad coverage of themes relevant to island biology and of different islands, archipelagos and ocean regions was a key feature of the conference. It was a strong indication that island biology is flourishing, and given the importance of Sherwin Carlquist's work across many research fields, it showed that his legacy lives on and grows. We will first provide a short overview of the life and work of the inspiring scientist to whom we dedicate the special issue, Sherwin Carlquist, before summarizing the main findings of each contribution in the special issue.

### Sherwin Carlquist's life and work

Sherwin John Carlquist was born in 1930 in California. From a young age, he was fascinated with islands, very likely due to reading some books about the Galápagos archipelago borrowed from Los Angeles School Library where Sherwin's mother worked. He received his undergraduate degree (1952) and a Ph.D. in Botany (1956) from the University of California, Berkeley. Following a postdoctoral stay at Harvard University, he began his teaching career at the Claremont Graduate School. In 1977, he also began teaching at Pomona College and continued working at both institutions until 1992. From 1984 to 1992, Carlquist was the resident Plant Anatomist at Rancho Santa Ana Botanic Garden. His last post was as an adjunct professor at University of California at Santa Barbara from 1993 to 1998.

Although trained as a wood anatomist, the early opportunity of doing research in Revillagigedo (Mexico) and Hawai‘i strengthened the allure of oceanic island floras to the young Carlquist. During his life, he has worked in many other island groups around the world, including Guadalupe, Society Islands, Samoa, Fiji, New Caledonia, Vanuatu, New Guinea, New Zealand, Australia, Taiwan, Japan, Borneo, Ryukyu Islands, Ogasawara (Bonin) Islands and the Subantarctic islands of New Zealand.

His scientific production includes >300 scientific articles, the majority being related to wood anatomy, as well as >10 books, 4 of them dealing with islands: *Island Life* (1965), *Hawaii: A Natural History* (1970), *Island Biology* (1974) and *Tarweeds and Silverswords: Evolution of the Madiinae (Asteraceae)* (2003). While all of these are very important contributions to our knowledge of island biology in general, and for Hawaiian natural history in particular, one of them, *Island Biology* (1974), became a major milestone in the field, comparable with classic books such as Darwin's *On the Origin of Species*, (1859), Wallace's *Island Life* (1881) or MacArthur and Wilson's *The Theory of Island Biogeography* (1967). In that book, Carlquist displayed, in an outstandingly organized structure, his encyclopaedic knowledge about the evolutionary trends and phenomena occurring among insular plants (and animals) and the selection pressures that drive them. The resulting set of evolutionary innovations is characteristic of island taxa and is known as the *island syndrome* (Adler and Levins 1994). Four of Carlquist's most important contributions to the development of the biology of island plants were as follows:

- *Long-distance dispersal to islands and secondary loss of dispersability on islands*. Following the classic ideas of Darwin, Wallace and Hooker, Carlquist was convinced of the importance of long-distance dispersal as a way to colonize oceanic islands and of the role of these as stepping stones between continents. In *Island Biology*, he formulated his famous 24 principles of dispersal and evolution (Midway and Hodge 2011), addressing the evidence for and implications of long-distance dispersal. He also focussed his attention on the several ways island plant (and animal) lineages, after arriving on an island via long-distance dispersal, lost or shifted their dispersal abilities when the ancestral trait was no longer adaptive. The increases in fruit size and weight, making the original fruit design no longer functional for dispersal (e.g. the Polynesian genus *Fitchia* of the Asteraceae—a subject on which Carlquist wrote his Ph.D. dissertation) or the flightlessness achieved by both birds and insects, are outstanding examples of this phenomenon.
- *Adaptive radiation*. Using his outstanding knowledge of island plants, Carlquist provided a comprehensive synthesis of information on the most important adaptive radiations of vascular plants on islands worldwide. He dedicated at least seven chapters of *Island Biology* to this subject, including a general introduction to the theme and detailed examples of evolutionary processes in Hawaii, Macaronesia, Galápagos, Juan Fernández, New Caledonia, New Zealand, islands off the coast of Western Australia and other islands.
- *Reproductive biology on islands*. In his book, Carlquist displayed his profound knowledge of floristic sexual systems providing plentiful examples of different reproductive systems among island plants (especially on Hawai‘i). He emphasized a general insular rule promoting outcrossing, i.e. self-compatible continental ancestors evolve different types of outcrossing on islands (dichogamy, herkogamy), via anemophily, heterostyly and all the possible stages in the transition from hermaphroditism to dioecy. He also elaborated upon hybridization in insular floras and its significance.
- *Insular woodiness*. Carlquist proposed that insular secondary woodiness is a result of evolution from continental herbaceous ancestors. He hypothesized that a release from seasonality occurred on islands—due to the buffer effect of the surrounding ocean—leading recurrently in different archipelagos (Hawai‘i, Galápagos, Macaronesia, etc.) and across many plant families to the *in situ* evolution of a woody habit from herbaceous plants. His view was in contrast to the traditional explanation by European island biologists for whom insular woodiness was not a derived feature but instead a basal characteristic of insular palaeoendemic species that became extinct on continents due to climatic or geological events after establishing on the islands. Only the emergence of phylogenetic analyses, a couple of decades after Carlquist's claims, made definitively clear that his interpretation was correct.

### Long-distance dispersal

The topics of more than a third of the papers covered in this special issue are related to the first three themes indicated above. Three papers advance our knowledge of different aspects of long-distance dispersal and its importance to island plant biology. **Alsos, Ehrich, Eidesen, Solstad, Westergaard, Schönswetter, Tribsch, Birkeland, Elven and Brochmann (2015)** present the first comprehensive study of long-distance dispersal to oceanic islands based on combined population genetic and floristic similarity analyses. The authors studied 25 representative vascular plant species of five Arctic Ocean islands (Greenland, Iceland and Jan Mayen) or island groups (Svalbard and Faroe), with the aim of shedding light on the origin and timing of their present floras' composition. They were able to detect that most plant species colonized those islands after the Last Glaciation through multiple long-distance dispersal events from several source regions, including Europe, Asia and North America, some of them travelling as far as 3000 km. The authors also found that the relative intensity of the founder effect was similar at the species and gene level, broadly corresponding with the predictions of the Island Equilibrium Theory (MacArthur and Wilson 1967), and indicating that species and genetic diversities on islands are shaped by similar processes. They report that insect-pollinated species show a strong founder effect that increases with island isolation and decreases with island size, whereas only a weak founder effect was found for wind-pollinated outcrossing species. Finally, they found that colonization patterns among the study islands were largely congruent, indicating that despite the importance of stochasticity, long-distance dispersal is mainly determined by the availability and geographic configuration of dispersal vectors. Another study showing that dispersal of species to oceanic islands from continental sources were not single events that occurred at random in the past is that by **Wolf, Rowe, Der, Schilling, Visger and Thomson (2015)**. By examining the morphological and genetic variation among bracken fern species (*Pteridium*) within the Galápagos Islands, these authors demonstrate that these processes can be dynamic and ongoing. The Galápagos archipelago sits on the equator ∼1000 km west of South America. Phylogeographic analyses show that *P. esculentum* from South America and *P. aquilinum* from the northern hemisphere have repeatedly colonized the islands in the past; *P. esculentum* populations have established numerous times, whereas *P. aquilinum* has not, but has resulted in the formation of hybrid combinations with *P. esculentum* (homoploid and allotetraploid) referred to as *P. caudatum*. This leads to speculation as to the origins, diversifications and evolutionary changes in other widely dispersed species inhabiting oceanic islands. In another study, **Vargas, Arjona, Nogales and Heleno (2015)** analysed the plant traits that allow long-distance dispersal and their effectiveness. These authors performed floristic and syndrome analyses on the native recipient flora of an Atlantic Ocean archipelago (Azores), finding that diplochorous species, i.e. those in which seed dispersal occurs by a sequence of two or more steps or phases, each involving a different dispersal agent, are over-represented relative to species in mainland Europe, but not when compared with their most likely propagule's source (mainland Portugal). They further analysed inter-island colonization patterns carried out in three oceanic archipelagos with well-studied floras (Azores, the Canaries and Galápagos) showing, as expected, a general trend of a higher number of islands colonized by vascular plant species with one or two long-distance dispersal syndromes than by unspecialized species. Nevertheless, statistical significance for differences in colonization was limited in some cases due to the low proportion of diplochorous species existing anywhere. Contrary to expectations, the authors assert that only a very marginal advantage for long-distance dispersal of species bearing multiple syndromes was observed.

### Adaptive radiation

Two papers in the special issue deal with adaptive radiation. Examples of adaptive radiation in oceanic islands due to colonization of new habitats distinct from the parent populations (e.g. cladogenesis) are abundant. However, diversification following colonization of similar habitats and accumulation of mutations eventually resulting in speciation (e.g. anagenesis) is also a common pattern among island floras (Stuessy *et al*. 2006). **Takayama, López-Sepúlveda, Greimler, Crawford, Peñailillo, Baeza, Ruiz, Kohl, Tremetsberger, Gatica, Letelier, Novoa, Novak and Stuessy (2015)** investigated the level of variation within and among species of five genera in the Juan Fernández Archipelago that demonstrate both cladogenetic and anagenetic speciation. Their key finding is that anagenetically derived species have populations with high levels of genetic variation yet no geographic genetic structure, whereas cladogenetic populations have less diversity within and among them. Their results also show important corollaries between island ages and levels of diversity. By examining multiple lineages, they demonstrate that these findings will be widely beneficial to the study of island evolution. Floras of many islands or island archipelagos are well studied and the species well known. However, it is not uncommon for new species to periodically be discovered. In some cases, new species are found during field surveys (Sporck-Koehler *et al*. 2015), and others may occur during taxonomic revision. The flora of the Cape Verde Islands consists of ∼740 species (∼12 % endemic) and has been well studied. **Romeiras, Monteiro, Duarte, Schaefer and Carine (2015)** demonstrated through molecular phylogenetic analyses of three endemic plant lineages on the Cape Verde Islands (genera *Cynanchum*, *Globularia* and *Umbilicus*) that additional species within each genus are in need of recognition. This study points out that the number of species in many of the plant lineages within this archipelago should be re-evaluated and the number of species within the flora, along with the level of endemism, is likely to increase substantially.

### Reproductive biology

Plant reproductive traits are dealt with in two papers, one by **Lord (2015)** and the other by **Watanabe and Sugawara (2015)**. The former reports the most comprehensive survey to date of reproductive traits of the floras of Subantarctic Islands—islands in the Southern Oceans such as the Falkland, Kerguelen or Macquarie islands. Southern Ocean islands have seen a steady increase in research interest, often related to large-scale habitat restoration and invasive species control efforts. Equally, both macroecological and experimental work on the reproductive biology of island plants have re-gained momentum, years after such questions had been at the core of Carlquist's work. Lord (2015) tests three interrelated classical hypotheses: whether self-compatibility is higher on islands as a result of self-fertile taxa being favoured as colonizers (Baker's law; Baker 1955); whether wind pollination is more common as a result of a pollinator-poor environment and whether gender dimorphism (dioecy) is also more frequent, resulting from selection against inbreeding risk in small island populations. The study reports indeed very high levels of self-compatibility (92.6 %, the highest percentage reported to date from any island flora), but found no clear trend for increased wind pollination or dioecy, partly because of the confounding factors of cold climates and trait frequencies in source floras. This highlights the importance of comprehensive data sets that include many islands across complete latitudinal and climate gradients and information from source floras for testing hypotheses in island biology (Kueffer *et al*. 2014). The study finds, however, strong indication that for many plant species, insect pollination is likely important, at least as part of a mixed strategy together with wind pollination, and thus, the work highlights the value of more detailed work on plant–pollinator interactions on these very isolated and cold Southern Ocean islands. Populations on oceanic islands are often small, and self-compatibility that can promote successful colonization may lead to inbreeding and the consequences associated with it. Island lineages have repeatedly evolved away from self-compatibility towards systems that promote outcrossing, including monoecy, dioecy, gynodioecy, androdioecy, heterostyly and other strategies. The evolution of heterostyly on oceanic islands is compounded by the complexity of the genetic system involved in maintaining it and, as such, is thought to be a rarity on remote island systems. Watanabe and Sugawara (2015) investigated the frequency of heterostyly among oceanic island lineages and then explore the evolution of this trait within *Psychotria* (Rubiaceae), a genus of ∼2000 species, widespread throughout the tropics and exhibiting variation for the heterostylous habit. Their study sheds light on the rarity of heterostyly on islands.

### Plant conservation

Many contributions in the special issue are highly relevant to plant conservation on islands. **Birnbaum, Ibanez, Pouteau, Vandrot, Hequet, Blanchard and Jaffré (2015)** present a very comprehensive data set on the small-scale distribution of over 700 native tree species on New Caledonia, which is in many ways unique for its very high species diversity, its complex geological and therefore biogeographic history and its habitats characterized by extreme, ultramafic soil substrates. This study reports two main conclusions. First, the habitat and environmental distribution of most of the species is broad, i.e. they occur across broad elevational, climatological and geological gradients, or to put it differently, they generally have broad ecological niches and are not specialists of one particular soil substrate or climate. Second, most species are highly spatially clustered, which the authors interpret as a result of reduced dispersability of island plants; an alternative or complementary explanation might be that this is at least partly a result of anthropogenic land use and resulting habitat fragmentation. Remnant native species distributions on many oceanic islands—or on islands that separated from continents millions of years ago—are nowadays confined to very small habitat fragments (Kueffer and Kaiser-Bunbury 2014). The results of Birnbaum *et al*. (2015) have important implications for plant conservation on such islands. It might be possible to conserve threatened species in a wide range of environmental conditions, but they will not themselves be able to overcome habitat fragmentation. Remnant fragments of high biodiversity must be strictly protected, and gene exchange between the last remnant stands restored through active conservation management. An interesting case study on the conservation biology of a particular threatened plant species in the Azores is provided by **Silva, Dias, Sardos, Azevedo, Schaefer and Moura (2015)**. These authors integrated information on the spatial distribution (including modelling of the environmental niche), population demography, conservation genetics and threats to a chamaephyte, *Veronica dabneyi*, assembling a comprehensive data basis for developing a conservation strategy for the species.

Understanding contemporary ecology and being able to plan appropriate conservation strategies requires an understanding of the past, and of how humans have shaped the modern biota. This is especially so for islands, where anthropogenic effects have often been recent and profound, causing extinctions and significant biological invasions (Carlquist 1974). In this special issue, **Shiels and Drake (2015)** show that a key factor in the rarity and extinctions of once-dominant endemic palms (*Pritchardia* spp.) of the Hawaiian Islands is predation of their seeds by rats—*Rattus exulans*, introduced by native Hawaiians at their arrival 750–800 years ago (Wilmshurst *et al*. 2011), and *R. rattus* introduced in the last 240 years. Identifying seeds as the most vulnerable stage of the palms' life history and rats as their main predator is a key step towards their conservation. Most tropical forest trees rely on animals for seed dispersal (Fleming and Kress 2013), yet the faunas of most tropical, oceanic islands have become depleted through extinction, extirpation and population decline following human colonization (Whitaker and Fernández-Palacios 2007). As a result, many island plant species may currently suffer from dispersal limitation (McConkey *et al*. 2012). Another contribution in this issue that shows the threats of seed dispersal disruptions is by **McConkey and Drake (2015)**, who demonstrate that flying foxes (*Pteropus tonganus*) in Tongan rain forests are the sole effective dispersers of 57 % of the plant species whose fruits they consume, and that they are especially important for large-seeded species. Combined with previous work which found that flying foxes are effective dispersers only when they forage at high densities (McConkey and Drake 2006), these new results reinforce the flying fox's role as the last remaining functional disperser for many tree species. The importance of seed dispersers for plant recruitment in islands is also highlighted by **Wandrag, Dunham, Miller and Rogers (2015)**. These authors compared the forest seed banks of a Micronesian island (Guam) that lacks seed dispersers with those of two islands where dispersers still exist. A key finding was that species in the seed bank were more likely to be found near adult conspecifics on the island without dispersers than on those with dispersers. The seed bank represents the viable pool of seeds in the soil from which new plants can recruit into plant communities, and its composition is determined by a combination of seed traits (e.g. germination, longevity and dormancy), the spatial pattern of seed dispersal and the actions of post-dispersal threats (e.g. pathogens and seed predators) (Fenner and Thompson 2005). Studying the seed bank's composition in disturbed sites/islands can provide us with useful information on the ultimate consequences of the loss of dispersers, predators, herbivores, etc. on plant species distribution and abundance.

### Habitat restoration

In recent years, the study of species interactions using network theory has proved to be an increasingly good framework to predict consequences of disturbance at a community level (Bascompte and Jordano 2013) and to advance conservation management. By using plant–pollinator networks and islands as model systems, **Kaiser-Bunbury and Blüthgen (2015)** identify a series of quantitative metrics to describe changes in network patterns that have implications for conservation. They distinguish between the ‘diversity’ and ‘distribution’ of interactions on three hierarchical levels (species, guild/group and network). Some of the metrics may be suitable indicators of anthropogenic changes in pollinator communities, and may allow assessment of the structural and functional robustness and integrity of ecosystems. They, thus, claim that a conservation network approach may be beneficial for advancing adaptive management. **González-Castro, Yang, Nogales and Carlo (2015)** used fleshy fruited plants and frugivorous birds in the Canary Islands to test which variables best explained the interactions in a frugivory network: the phenotypic match between bird and fruit traits, or the relative abundance of birds and fruits. The most important explanatory variables were phenotypic, especially the overlap between fruit size and bill width, but fruit and bird abundance also played a significant role. This study provides a useful model for examining mutualistic networks in more diverse and complex systems. Ecological networks are a good tool to understand how alien species infiltrate the receptive communities as well as how and to what extent they can impact and modify the structure of such communities. **Traveset, Chamorro, Olesen and Heleno (2015)** show that alien plant species integrate easily into the native communities in the Galápagos and that the impact on the overall network structure is rather low, except for an increase in network selectiveness (i.e. species become more selective in their partner's choice, interacting with species that tend to be less abundant). Following a study on the emergence of novel communities in Galápagos (Traveset *et al*. 2013), these authors find that aliens represent a high fraction (>50 %) of the total number of interactions, and that they prevail in all habitats and seasons. In spite of the low overall effect on network structure, they suggest that aliens may well have an important effect on community functioning as they may influence the reproductive success of native species. When native species are lost, their functional role in the community may or may not be replaced by alien species. In a study carried out in Hawai‘i, **Pejchar (2015)** found that introduced birds can act as seed dispersers of native plants replacing—but only to a limited extent—a native frugivorous bird (‘ōma‘o, *Myadestes obscurus*) in areas in which it has become extinct. This author claims that alien birds are imperfect substitutes for native species and that seed dispersal patterns actually are notably altered following local extinctions of ‘ōma‘o, and suggests reintroducing it to suitable habitats within its historical range.

Species losses and gains result in major shifts in ecosystem composition and function (Wardle *et al*. 2011), and this presents challenges for restoring island ecosystems. Island biologists are increasingly considering reintroducing ecologically important species to suitable habitats within their historic range and to neighbouring islands with depauperate communities of vertebrate seed dispersers. **Hansen (2015)** is one such study advocating ‘rewilding’ introductions of large non-native tortoises to small areas of islands where large native herbivores have been extinct for hundreds of years: flightless birds such as moa-nalo in Hawai‘i and giant native tortoises on Rodrigues and Round Island in the Mascarene Islands. On these islands, attempts to restore native ecosystems are difficult because of multiple invasive alien plants that can out-compete native plants. In his study, Hansen presents preliminary data to show that native woody plant growth benefits when non-native large tortoises graze sites. On this basis, he proposes carefully monitored introductions of large tortoises worldwide to islands where large herbivores are extinct as a tool to assist the restoration of degraded island ecosystems.

Palaeoecological studies can help establishing whether plant species have been introduced to islands. In the Galápagos Islands, pollen evidence from before human settlement revealed that plant species that had been considered introduced to the islands were in fact native (van Leeuwen *et al*. 2008). In contrast, pollen evidence from the Subantarctic Auckland Islands, south of New Zealand, shows that a tree, *Olearia lyallii*, is not native to the islands (**Wilmshurst, McGlone and Turney 2015**). This species first appears in the pollen record in the early 19th century, when it was probably inadvertently introduced by sealers from the Snares Islands, further to the north. Historical paintings and photographs during a period of settlement on the Auckland Islands in the mid-19th century show areas of forest clearance and burning, and the authors of this contribution claim that these are the areas where the tree has become locally dominant up to the present.

## Concluding Remarks and Future Directions

The First International Island Biology conference in 2014 in Honolulu and the resulting special issue demonstrate that islands are reasserting their role as ideal model systems for the latest research questions and methods in ecology, evolutionary biology, biogeography and conservation. The key research questions related to long-distance dispersal, adaptive radiation and plant reproductive systems, broadly covered 40 years ago by Sherwin Carlquist in his classic book, *Island Biology*, are now being addressed with much more comprehensive data and new methodologies. While island biology might indeed be entering a new golden era of research (Fernández-Palacios *et al.* 2015), we are also at the brink of losing these unique biological systems for research and humanity. Literally, thousands of endemic island species survive as only a few individuals or small and fragmented populations—and for most of them, very little is known about their basic biology. What is unequivocally clear, however, is that most of these species will disappear from the wild in this century unless we markedly intensify our conservation efforts.

## Contributions by the Authors

A.T. led the writing but all authors contributed similarly to this article.

## Conflict of Interest Statement

None declared.
